# FOOD INSECURITY AND COST-RELATED MEDICATION NON-ADHERENCE IN OLDER ADULTS: A SYSTEMATIC REVIEW

**DOI:** 10.1093/geroni/igz038.2364

**Published:** 2019-11-08

**Authors:** Lisa L Boss, Shaunna Caouette

**Affiliations:** UTHSC, Houston, Texas, United States

## Abstract

Food insecurity (FI) is defined as having limited access to nutritional and safe foods due to lack of financial resources and is believed to negatively influence health outcomes. Older adults, in particular, face rising healthcare costs and may be forced to choose between purchasing prescribed medications and using their limited financial resources for basic needs, such as food. The purpose of this systematic review was to examine the relationship of food insecurity (FI) and cost-related medication non-adherence (CRN) in older adults living in the community setting. A comprehensive, electronic review of the literature was performed. Criteria for inclusion were original quantitative or qualitative research, written in English, human participants ≥60 years, and published from January 2000 through January 2019. The total number of studies included was six. Main findings from the studies largely indicate that FI and CRN are significantly and positively correlated in older adults living in American communities. Further, CRN increases with the severity of FI. Most participants in these six studies were female, non-Hispanic white, with an annual household income <20k, and with less than a high school education. Although preliminary evidence is promising, research with more rigorous design is warranted to better understand the relationship of FI and CRN in older adults, and to develop appropriate interventions and programs for this growing public health concern.

